# First description of subglacial megalineations from the late Paleozoic ice age in southern Africa

**DOI:** 10.1371/journal.pone.0210673

**Published:** 2019-01-30

**Authors:** Graham D. Andrews, Andrew T. McGrady, Sarah R. Brown, Shannon M. Maynard

**Affiliations:** Department of Geology and Geography, West Virginia University, Morgantown, West Virginia, United States of America; Chinese Academy of Geological Sciences, CHINA

## Abstract

We identify late Paleozoic ice age (LPIA) subglacial megalineations from field and geospatial imagery of the Twyfelfontein area of northern Namibia, and present the results of a geomorphometric analysis of those data. Asymmetric 0.1–1.5 km-long megawhalebacks indicate a paleo-ice flow to the northwest. We infer that an ice stream draining the LPIA Kaokoveld ice sheet existed within the proto-Huab River valley and that was comparable to ice streams in modern Antarctica. Recognition of a paleo-ice stream in northern Namibia supports interpretations of glaciogenic sedimentary successions (Itararé Group) in southern Brazil that suggest the presence of major, terrestrial glacial outlet systems in southern Africa during the LPIA.

## Introduction

We infer the presence of a late Paleozoic ice age (LPIA) paleo-ice stream in the Huab River valley based on the occurrence, shape, and size of elongate bedforms exposed on a Carboniferous-Permian glacial erosional surface. Many parts of Gondwana exhibit bedrock features including striations and glacial valleys formed during the LPIA, including Argentina [1], southern Australia [2], southern Brazil [3], Ethiopia, [4], Namibia [5, 6], South Africa [7, 8], and Uruguay [9]. Evidence for ice streams draining the LPIA ice sheets, however, is reported only rarely and mostly from the peripheries (Chad [10], Ethiopia [4]), but never before from southern Africa despite being at or close to the South Pole throughout the Carboniferous and early Permian [11].

Ice streams are channels filled with ice flowing faster than the surrounding ice sheet, and are the principle arteries for ice flow from the sheet’s center to the margin [12], often forming flow networks ≤10^3^ km in length and with catchments of ≤10^6^ km^2^ [13]. ‘Megalineations’ result from deposition, deformation, or erosion by the overriding ice stream where the degree of elongation is proportional to the velocity of the overriding ice mass (e.g., [14]). Mega-scale subglacial lineations (MSGLs) are the largest and most elongate megalineations, and are closely associated with modern fast-flowing (>10^2^ m a^-1^) ice streams over soft-sediment in Antarctica (e.g., [15]) and Greenland.

Megalineations form a size-shape continuum from meter-scale roche moutonées, through rock drumlins and megawhalebacks, to kilometer-scale MSGLs [16]. Because they are bedrock features, they have a high preservation potential through repeated glacial advances and retreats [17], and are reliable indicators of paleo-ice flow directions. Pleistocene megalineations have been used extensively to reconstruct paleo-ice streams in Antarctica, Canada, and Scotland [18], and provide a wealth of understanding about ancient ice dynamics and ice extents.

## Megalineations in the Twyfelfontein area

**Fig 1:**
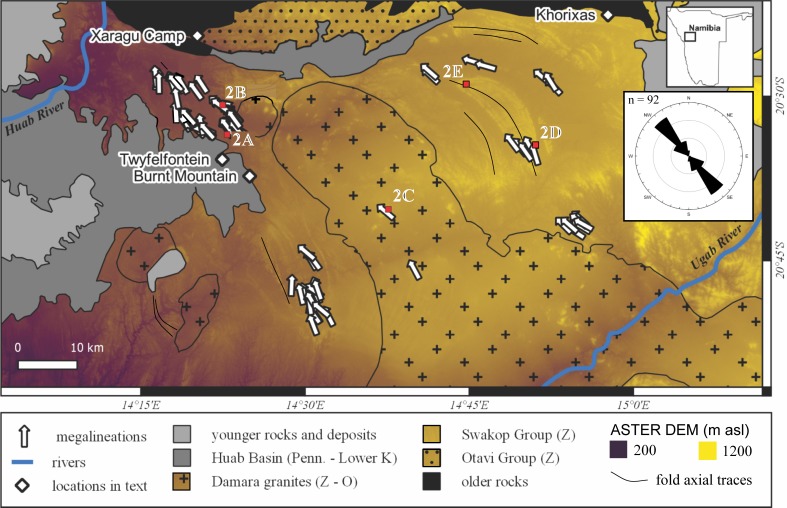
Simplified geological map of the Twyfelfontein area highlighting the distribution of Damaraland basement rocks and present-day topography (false-colored ASTER digital elevation model). The trends and inferred ice flow directions of MSGLs are shown as white arrows in the inset circular histogram. The axial traces of selected large folds are shown in black. The location of images in Fig 2 are shown. Geology from the Digital Atlas of Namibia (http://www.uni-koeln.de/sfb389/e/e1/download/atlas_namibia/index_e.htm).

The Twyfelfontein area of Kunene Region, NW Namibia (Fig 1) encompasses the southeastern margin of the Permian–Early Cretaceous Huab Basin, an outlier of the Karoo Supergroup, and the underlying Proterozoic Damaraland basement of folded metaturbidites (Swakop Group) intruded by voluminous granitoid plutons. The modern Huab River is misfit and follows a broad, southwest-trending LPIA glacial valley identified by Martin [19] that contains small, isolated outcrops of Pennsylvanian–early Permian Dwyka Group diamictite overlain by locally thick sections of the Verbrandeberg (fluvial) and Tsarabis (deltaic) formations of the middle Permian to early Triassic Ecca Group [20, 21].

**Fig 2:**
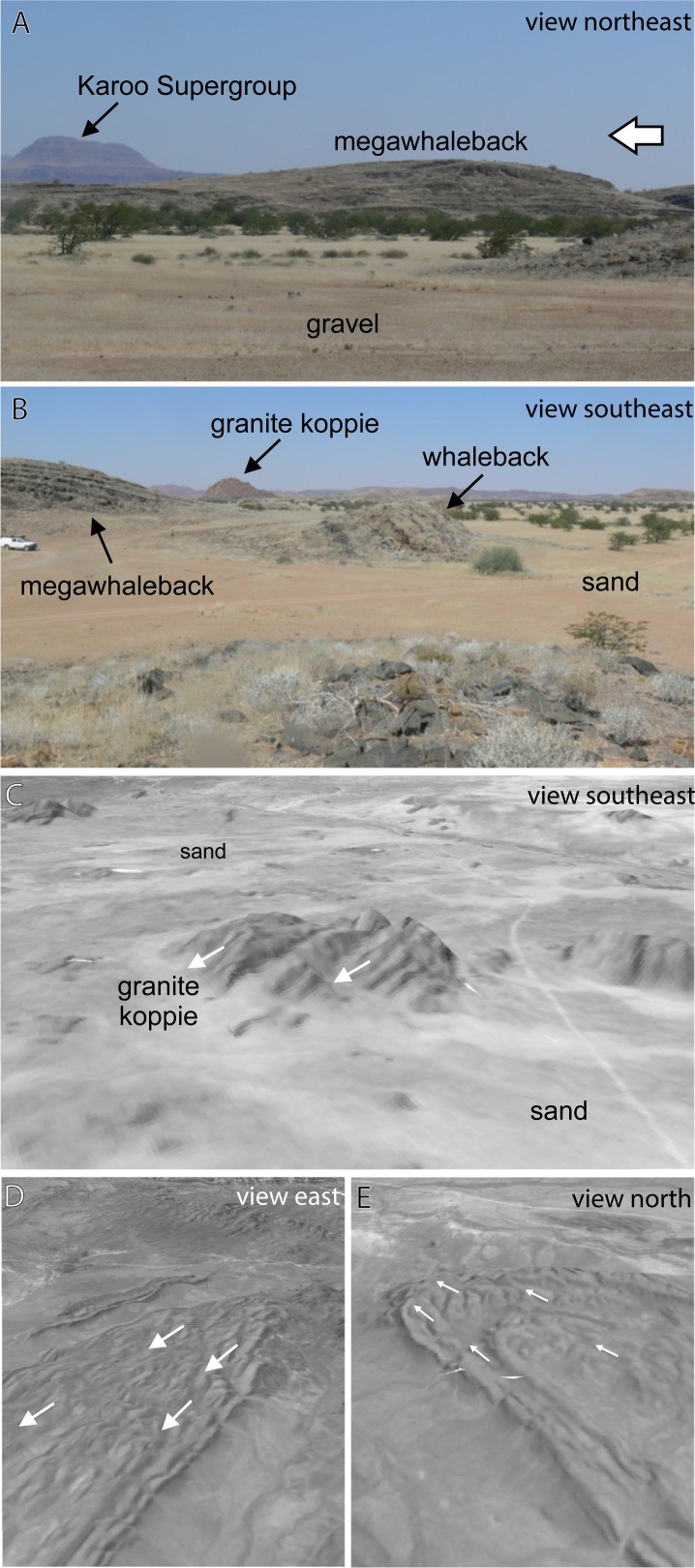
Megalineations at Twyfelfontein. (A) Side-on field photograph of an asymmetric megawhaleback at Twyfelfontein, inferred ice flow direction from right (SE) to left. The megawhaleback is approximately 15 m tall. (B) Along axis view of a whaleback and megawhaleback pair near Xaragu Camp, truck for scale. The axis of the whaleback in the middle ground is parallel to the strike of the southwest dipping beds. The axis of the neighboring megawhaleback is perpendicular to bedding at the fold hinge (extreme left above truck). Note loose flaggy regolith on whaleback surface in the foreground. (C) Oblique image of megawhalebacks in a large granite koppie between Burnt Mountain and the Ugab River; USGS EROS (Earth Resources Observatory and Science (EROS) Center) http://eros.usgs.gov/#; illustrative of copyrighted images used in this study. (D and E) Oblique images of the southern and northern closures, respectively, of a large fold in the Swakop Group; USGS EROS (Earth Resources Observatory and Science (EROS) Center) http://eros.usgs.gov/#; illustrative of copyrighted images used in this study.

Megalineations sculpted into the Damaraland basement are exposed where the Dwyka and Ecca groups have been eroded away exposing the late Paleozoic buried erosional landscape (Fig 2A). There the megalineations are buried directly beneath carbonaceous shales and siltstones of the glaciogenic Dwyka Group (e.g., Burnt Mountain). The exposed bedforms are typically NW-trending (Fig 1), and often parallel to the strike of the folded, steeply-dipping, greenschist-facies metaturbidites of the Swakop Group [22]. Resistant siliciclastic and calc-silicate metaturbidites are intercalated; positive and negative megalineations do not appear to be controlled by differences in lithology. Some positive megalineations parallel upright fold hinges (Fig 2B), but most are carved into fold limbs. Polished surfaces and striations were not observed on the five megalineations visited; however, these features were heavily karst-weathered (dissolution pits, bedding parallel ‘clints’ and bedding perpendicular ‘grikes’; Fig 2B). We infer that any glacial polish or striations that were present have been removed by weathering.

Analysis of freely available satellite imagery in Google Earth Pro reveals the presence of at least 93 asymmetrical megalineations in the Twyfelfontein area (Fig 1) including the approximately dozen observed on the ground between Twyfelfontein (20°34’07”S 14°22’15”E) and Xaragu Camp (20°24’07”S 14°20’07”E; Fig 2A & 2B). The area between Twyfelfontein and Xaragu Camp is open, uninhabited savannah: no permissions were required for access and no protected species were interfered with. The bedforms occur in strings, as isolated hills, or as clusters; in all cases the positive megalineations are emergent from the surrounding desert surface and the negatives are filled by modern sand and gravel. Positive and negative bedforms form two clusters within large koppies in an adjacent Damaraland granite pluton (Fig 2C).

The positive megalineations are consistently asymmetric, in the field and in the digital elevation model, where the highest point is at the SE-end (e.g., [16, 18]). This is consistent with regional paleocurrent directions to the west established from glacial striations and sedimentary structures in the Dwyka Group [23].

The megalineations parallel the strike of bedding in the Swakop Group everywhere except around the closures of a kilometer-scale, doubly-plunging synform south of Khorixas (Fig 1). The asymmetrical bedforms are consistently parallel and trend NW-SE, even around the tight fold closures where the bedding strike varies through ~320° (Fig 2D and 2E). Newton et al. [17] emphasize the importance bedrock structure on the orientations of many bedforms, and note the rarity of wholly independent orientations. However, the parallelism of these megalineations around basement structures and across different lithologies suggests that their orientations are independent of the lithological and structural grain of the Swakop Group. Furthermore, the scale of the bedforms is significantly greater than typical thicknesses of different lithologies (≤5 m) within the Swakop Group (e.g., Fig 2B). We cannot rule-out that the bedrock grain has had a minor influence on the orientation of megalineations, conversely it seems likely that linear sculpting will be facilitated and enhanced when the ice-flow direction and bedrock grain are coincident. We infer that the structural grain of the bedrock plays a second important role in exaggerating the shape and size of the bedforms at Twyfelfontein, making them more prominent and easier to identify.

## Geomorphometric analysis

We measured to maximum lengths and widths of 93 megalineations around Twyfelfontein using freely available satellite imagery in Google Earth Pro (Fig 1, S1 Dataset). Axial lengths were measured in GE Pro using the built-in line measuring tool. The elongation ratio (E) is calculated as the length of the long axis divided by the perpendicular short axis length. To test our methodology and the veracity of GE Pro-sourced data, we conducted a blind analysis of different types of megalineations sculpted into sedimentary or metasedimentary rocks reported in Krabbendam et al. [18]. These include the Cree Lake drumlin field (northwest Saskatchewan), as well as ‘megagrooves’ at Amadjuak Lake (southern Baffin Island) and near Ullapool (northern Scotland).

**Fig 3:**
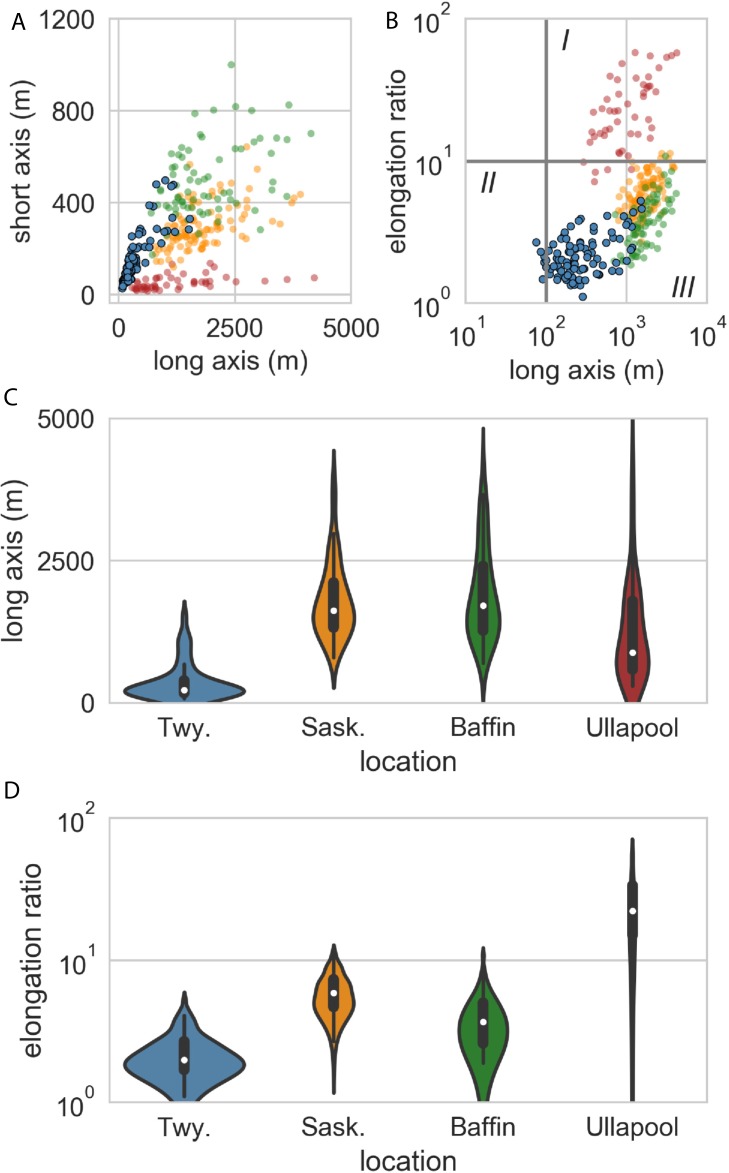
Geomorphometric data. (A) Cross-plot of MSGLs axes from Twyfelfontein (blue), Cree Lake, Saskatchewan (orange), Amadjuak Lake, Baffin Island (green), and Ullapool, Scotland (red). (B) Cross-plot of long axis length and elongation ratio. Fields for (I) megaridges, (II) rock drumlins and whalebacks, and (III) megawhalebacks follow Krabbendam et al. [18]. (C) Violin plot of the distribution of megalineation long axes between Twyfelfontein and the three comparison sites. The white dot is the median value, the heavy black line is the interquartile range, and the ‘violin’ is a mirror-imaged kernel density plot. (D) Violin plot of the distribution of megalineation elongation ratios for Twyfelfontein and the three comparison sites.

The bedforms at Twyfelfontein inhabit a narrow range of lengths (75–1,523 m; Fig 3A), widths (28–499 m), and E values (1.1–5.3; Fig 3B). The median long axis length is 226 m, the median width is 117 m, and the median E value is 2.0. Using the classification scheme of Krabbendam et al. [18] 88 of the Twyfelfontein bedforms are ‘megawhalebacks’ (long axis >100 m, E <10; Fig 3B) and five are ‘whalebacks’ (long axis <100 m, E <10).

The bedforms in Saskatchewan, Baffin Island, and Scotland are generally longer (Fig 3C) and more elongate (Fig 3D) than at Twyfelfontein; although the Namibian examples are part-buried in modern sand, and therefore their dimensions are minima. Those in the Canadian examples are classified as megawhalebacks, whereas those at Ullapool are more elongate ‘megaridges’. This range of morphologies is typical of megalineations where there is a continuum between different bedforms (e.g., [16]), and especially between drumlins and whalebacks (<100 m long and E <0.1), megawhalebacks (>100 m; E <10), and megaridges (>100 m; E >10).

## Implications

### A LPIA paleo-ice stream?

The megalineations at Twyfelfontein, and associated tillites of the Pennsylvanian-Permian Dwyka Group, record the existence of a significant body of ice that flowed to the (modern) northwest, probably during the LPIA. There were probably numerous advances and retreats of ice during the LPIA, and we do not attempt to ascribe these features to a specific glacial event.

The overwhelming majority of Twyfelfontein bedforms are megawhalebacks [18] (Fig 3B) and would fall across the rock drumlin and MSGL fields of Ely et al. [16] and the rock drumlin field of Stokes et al. [24]. The bedforms described meet some or all of the criteria used in previous studies to classify MSGLs and to identify paleo-ice streams. MSGLs are typically differentiated from other elongate bedforms by E values of >10 although statistical analysis of very large geospatial datasets (*n* > 10^4^ bedform features) suggests an elongation value >8 is more reasonable where a continuum exists with drumlins [16, 24]. In Stokes et al. [24]’s study only 23% of megalineations had E values >10. Krabbendam et al. [18] break-out highly (E >10) and medium (E <10) elongation megalineations, where medium elongation megalineations are transitional with and occur with typical drumlins. Recognizing that there is (1) a continuum in bedform shape and size [16], that (2) megalineations and drumlins often coexist, and (3) that the most elongate MSGLs are found in soft, marine sediments (e.g., [14]), an arbitrary definition of minimum MSGL shape and size is not useful in ancient, eroded, and poorly exposed glacial landscapes.

We infer that the megalineations at Twyfelfontein can be considered MSGLs or as transitional between elongate drumlins and MSGLs. Therefore, they record passage of an ice stream of some significant ice flow velocity. The relationship between ice stream velocity and underlying bedforms is limited to geophysical studies of active ice streams where the velocity is known (e.g., [15]). All things being equal, drumlins and megawhalebacks (E <10) are interpreted to form at 10^2^–10^3^ m a^-1^, and MSGLs (E >10) at >10^3^ m a^-1^ [24]; rates comparable to many Antarctic ice streams. Therefore, it is likely, that similar conditions have existed under comparably sized, long-lived ice sheet(s) in the LPIA of Gondwanaland, and that the megalineations at Twyfelfontein preserve a record of this ice streaming.

### Glacial outflow from the Kaokoveld ice sheet

**Fig 4:**
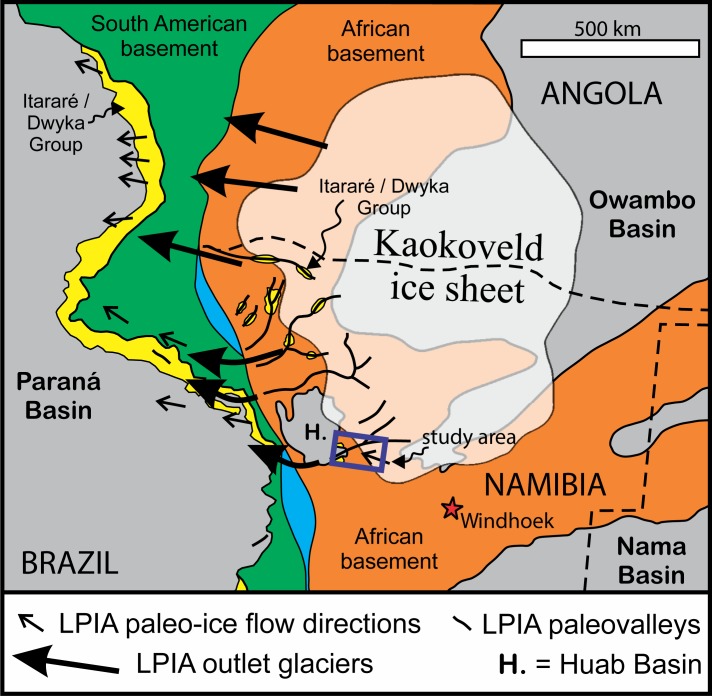
Paleogeographic reconstruction. Reconstruction of the SE Brazilian and southern African margins during the LPIA, adapted from [3] and [27], and with data from [19] and [20]. Known paleocurrent directions preserved in the Itararé Group and the paleo-ice flow directions reported here are shown.

Northern Namibia and then-adjacent parts of what is now SE Brazil were located close to the South Pole in Early Mississippian and moved to about 60°S through the Pennsylvanian and Permian [11]. The region, along with the rest of southern Africa, was inundated by LPIA ice lobes in the Middle and Late Mississippian and again from the Early Pennsylvanian to the earliest Permian [6]. The ice sheet over northern Namibia (‘Kaokoveld ice sheet’ [3]; Fig 4) probably waxed and waned throughout the LPIA, and may sometimes have been contiguous with the adjacent Namaland and Transvaal ice sheets (e.g., [25]). Ice over northern Namibia and southern Angola flowed west towards outlets that supplied glaciogenic sediment to the Paraná Basin in Brazil (Fig 4; e.g., [26]) whereas ice over central and southern Namibia outlet to the Kalahari, and possibly the Karoo, basins to the south and southeast (e.g., [7]).

There is abundant evidence of LPIA sediment supply from southern Africa in the Itararé Group, contemporaneous with the Dwyka Group, within the Paraná Basin. The Itararé Group records glacial advance and retreat at the ice margin followed by the onset of open marine conditions [26]. Northwest-trending paleochannels and paleovalleys, including sculpted bedrock surfaces, have been described throughout the Itararé Group [3], where paleocurrent directions are consistently northwesterly (Fig 4; [26]). The scale of the paleovalleys present in the Itararé Group (0.8–8 km wide, >65 km long; e.g., [28]) and the bedforms within them indicates a sustained, voluminous glacial discharge, suggesting that they were downstream of major ice streams.

Plate reconstructions juxtapose SE Brazil and northern Namibia during the LPIA supporting links between the Kaokoveld ice sheet and its distal deposits in the Paraná Basin (e.g., [3, 23, 26]). Hitherto, the evidence of LPIA ice streams has come from downstream glaciogenic sedimentary records in the paleovalleys of the Itararé Group. Our observations from immediately below the contemporaneous Dwyka Group provide the first definitive evidence of ice streams from the Namibia highlands that likely fed the Itararé Group paleovalleys. If this is correct, then ice flowing through the paleo-Huab River valley at Twyfelfontein continued for at least another 200 km downstream before grounding and calving into the seas of the Paraná Basin (Fig 4).

## Summary

We have identified a field of large, subglacial sculpted bedforms in northern Namibia that lead us to infer the past existence of major ice stream draining a LPIA ice cap in southern Africa. The location and paleo-ice flow directions of the whalebacks and megawhalebacks indicate northwestward flow of ice along the paleo-Huab River valley that likely discharged into the shallow marine environment several hundred kilometers downstream in modern Brazil.

## Supporting information

S1 Dataset MS Excel file containing megalineation length (m), elongation ratio, and azimuth from the Twyfelfontein area. (XLSX)
